# High Prevalence of Arterial and Venous Corona Mortis Variants in Cadaveric Dissection

**DOI:** 10.7759/cureus.98303

**Published:** 2025-12-02

**Authors:** Diego Álvarez-Manilla-Cruz, Adelina Rojas-Granados, Pablo Betanzos-Madrigal, Ian A Zavala-Ramos, Aurelia Martínez-Díaz, Erandi Ugalde-Santos, Esteban M Arellano-Rivera, Julio Pérez-Alavéz, Manuel Angeles-Castellanos

**Affiliations:** 1 Department of Innovation in Human Biological Material, Faculty of Medicine, Universidad Nacional Autonoma de Mexico, Mexico City, MEX; 2 Department of Anatomy, Faculty of Medicine, Universidad Nacional Autonoma de Mexico, Mexico City, MEX; 3 Hospital of Traumatology, Orthopedics, and Rehabilitation, Unidad Médica de Alta Especialidad "Dr. Victorio de la Fuente Narváez" del Instituto Mexicano del Seguro Social, Mexico City, MEX; 4 Orthopedic Service, Grupo Angeles Hospital, Mexico City, MEX; 5 Departament of Anatomy, Faculty of Medicine, Universidad Nacional Autonoma de Mexico, Mexico City, MEX

**Keywords:** cadaveric study, hip fractures, pelvic surgery, vascular anatomy, vascular variation

## Abstract

Background: The arterial or venous corona mortis (CM) is an anatomical vascular variant found in the retropubic area. It is an anastomosis between the external iliac system and the obturator vessels. This study focuses on morphometric analysis and the establishment of the prevalence of arterial and venous CM in the Mexican population.

Methodology: A total of 108 hemipelves from human cadavers embalmed with a propylene-glycol solution were analyzed using a suprapubic approach. The abdominal viscera were mobilized to locate the common iliac arteries and veins, which were perfused with red latex for arteries and blue for veins. The vessels of the internal iliac system were identified and dissected, and their morphological characteristics, pattern of variation, and laterality were examined. Measurements included caliber, length, and distance between the CM and the pubic symphysis, using an electronic Vernier caliper for all measurements. Each measurement was performed in triplicate and is presented as mean ± standard error.

Results: Of the studied hemipelves, 14 were female, and 94 were male. A morphometric analysis was performed, where we found that 32 (29.6%) of the hemipelves did not present any arterial variants and only 11 (10.1%) did not show a venous variant. Accordingly, this study found a prevalence of 76 (70.4%) for arterial variants and 97 (89.9%) for venous variants.

Conclusions: The frequency of CM had not been reported in the Mexican population; however, we found a higher prevalence (48%) of CM than previously reported (36%).

## Introduction

The term *corona mortis* (CM) means crown of death, and it serves as a warning to the surgeon that, if accidentally damaged, profuse and uncontrollable bleeding can be life-threatening. CM is a vascular anastomosis between the external and internal iliac arteries or veins. However, the severity or clinical significance depends on the caliber and length of the vessels [1].

The obturator artery originates from the internal iliac artery and runs through the obturator canal toward the medial femoral artery into the medial compartment of the thigh; the same pattern is often seen in veins [2]. Interestingly, there is also the possibility that the obturator artery does not originate from the internal iliac system, but rather from the external iliac artery. In this case, it is referred to as an aberrant obturator artery or vein. Any vessel originating from the external iliac system and entering the obturator foramen without anastomosis with the internal iliac system is considered aberrant, replacing the obturator artery or vein that usually originates from the internal iliac system [3,4].

On the other hand, the existence of an accessory obturator artery has been described, usually originating from the external iliac artery, which generally originates together with the inferior epigastric artery from a common trunk and enters the obturator canal without anastomosis with the main obturator [5].

An anastomotic artery can also be found, originating from the inferior epigastric artery or directly from the external iliac artery, which joins the obturator artery. This anastomotic bridge is what we refer to as the CM artery [4].

The CM artery or vein is a vascular structure located in the retropubic region and curves above and behind the superior ramus of the pubic bone pubic at variable distances from the symphysis. It anastomoses with the obturator artery [6,7].

The clinical relevance of the CM artery morphometry is established by the vascular variation in size, shape, and incidence, which can vary significantly between individuals and populations. This variation has implications for both normal function and certain pathological conditions, particularly those related to surgical [8].

Due to the clinical importance of the CM artery, attempts have been made to characterize it morphologically and topographically. Some reports indicate its location in a retropubic position, at the level of the superior ramus of the pubic bone and at a highly variable distance (40-96 mm) from the pubic symphysis [2,9]. The prevalence has been reported to vary depending on the population and study method, with venous CM being the most common, at 41.7%, while arterial CM is the most prevalent, at 17.0% [10]. Therefore, understanding the morphometry and incidence of the CM artery allows surgeons to anticipate procedural challenges in this region and predict surgical outcomes with greater confidence, since heavy bleeding and posterior vasoconstriction in the pelvic cavity during pelvic fractures involving the quadrilateral plate and the obturator foramen can potentially damage the CM, or surgical procedures involving the anteroinferior abdominal wall or other procedures involving the retropubic region.

This study focuses on the morphometric analysis of the CM artery and vein, aberrant obturator vessels, and accessory obturator vessels, identifying surgically significant variants and establishing the prevalence of the CM artery in the Mexican population.

## Materials and methods

Ethical considerations

All human bodies used in this study were obtained from the institutional body donation program (BDP) of the Faculty of Medicine of the National Autonomous University of Mexico (UNAM) and the Institute of Forensic Sciences of Mexico City (INCIFO) between 2023 and 2024, through an agreement signed on March 7, 2011, between this institution and UNAM. All procedures and handling of biological material carried out for this study complied with the established standards and the Mexican Federal Health Law for the control and sanitary disposal of human tissues and cadavers, Chapter V (DOF March 26, 2014), as well as adhered to international regulations on the care and ethical use of corpses [11]. The protocol for this study was approved by the Institutional Ethics and Research Committee of the Faculty of Medicine, UNAM, under registration number UNAM-FM/DI/083/2023, on May 24, 2023.

Study design

This is a cross-sectional, descriptive study. The sample consisted of 108 hemi-pelvises from 54 human cadavers, 47 male and 7 female, ranging in age from 29 to 91, with a median of 55 years and an average of 55.85 years, with intact pelvic cavities. These cadavers were kept under the protection of the Department of Innovation in Human Biological Material, Faculty of Medicine, UNAM. Exclusion criteria included cadavers that presented pelvic vessel laceration during the autopsy or prior dissection. Cadavers of subjects with infectious diseases (confirmed or reasonably suspected clinically), including human immunodeficiency virus (HIV) infection or hepatitis, were also excluded.

Preservation and dissection

**Figure 1:**
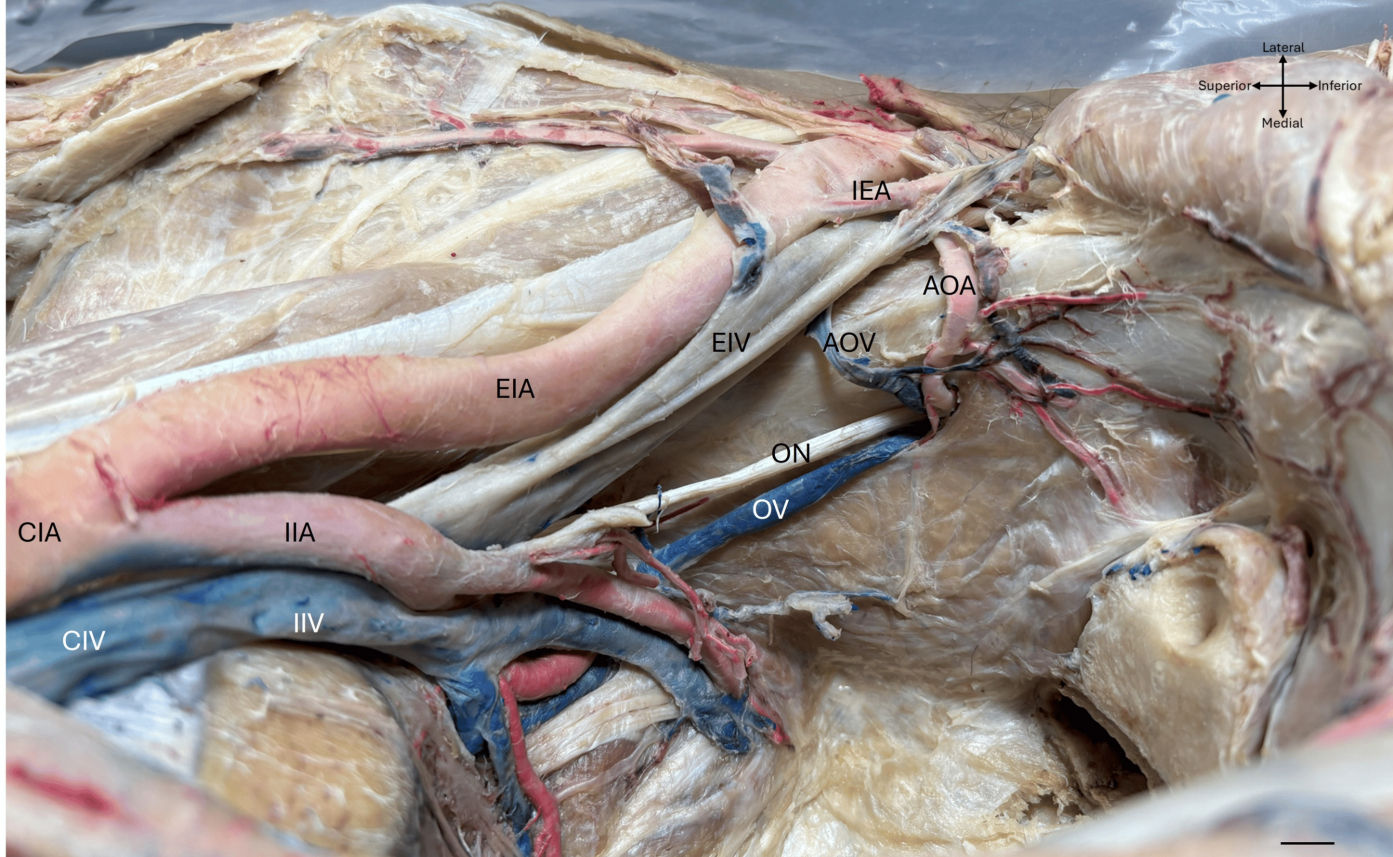
Arrangement of the pelvic vessels showing an aberrant obturator artery (AOA) and an accessory obturator vein (AOV), along with the external iliac vein (EIV) and the obturator vein (OV). The cross-arrows indicate the orientation of the body, and the scale bar represents 1 cm. CIA, common iliac artery; EIA, external iliac artery; IIA, internal iliac artery; IEA, inferior epigastric artery; CIV, common iliac vein; EIV, external iliac vein; IIV, internal iliac vein; OV, obturator vein; ON, obturator nerve

All bodies analyzed were embalmed with a solution of 80% propylene glycol, 15% isopropanol, and 5% formaldehyde. For dissection, a medial suprapubic incision was used. The abdominal viscera were mobilized to locate the common iliac arteries and veins, which were perfused through the inferior vena cava and the abdominal aorta at the level of L4, with blue and red latex, respectively (LATEX, Lot:240907; Polyforms de Mexico) (Figure 1).

Subsequently, all the vessels of the internal iliac system were identified and dissected to determine their morphological characteristics and precise dimensions. The pelvic viscera were then removed, specifically the colon, rectum, and urinary bladder, as well as the uterus in female cadavers.

Definition of the Variants

We used the classification proposed by Kostov et al. [4]: (1) CM, an artery or vein that anastomoses the external iliac system with the internal iliac system; (2) aberrant obturator, an artery or vein originating from the external iliac system and entering the obturator foramen, without anastomosis with the iliac vessels; and (3) accessory obturator, an artery or vein originating from the external iliac system, without anastomosis with the internal iliac vessels.

Measurements

**Figure 2:**
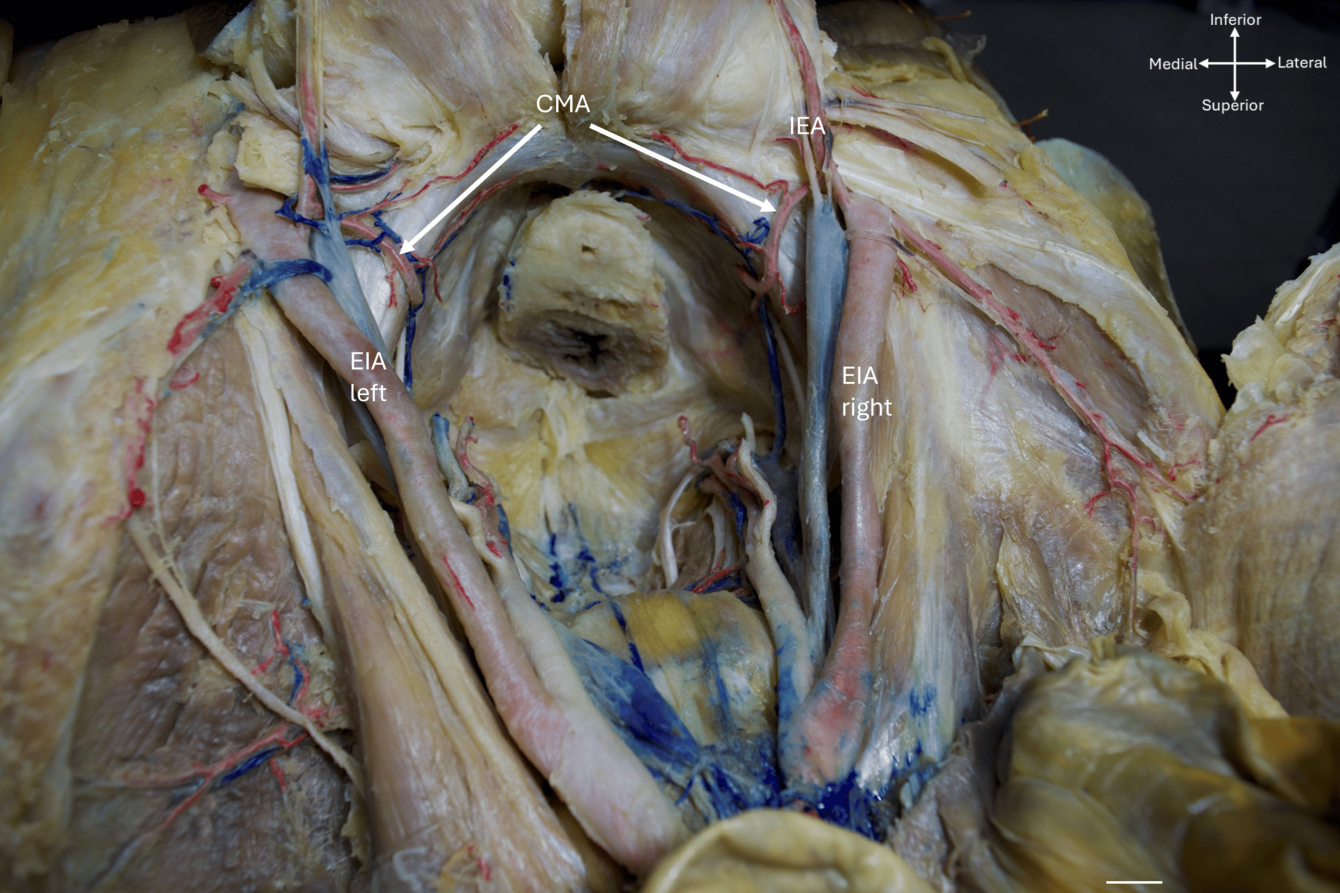
Bilateral corona mortis artery (CMA) with right and left external iliac artery (EIA) and inferior epigastric artery (IEA). The cross-arrows indicate the orientation of the body, and the scale bar represents 1 cm.

Once the vessels of interest were exposed, the pattern of variation and the laterality of the vascular variant were determined (i.e., whether it appeared bilateral, unilateral on the right, or left; Figure 2). Measurements were taken of vessel length from their origin to their termination or to the obturator canal, as well as vessel caliber and the distance from the CM artery to the pubic symphysis. All measurements were performed using an electronic vernier caliper and micrometer, and all values were expressed in millimeters (mm). Each measurement was performed by three different researchers, all of whom had been previously trained to ensure consistency and stability of the results. The three measurements for each variable were averaged to obtain the value for each hemipelvis.

Analysis

The average of all measurements was calculated and presented as the mean ± standard error (SE). Comparisons between structures (veins versus arteries) were performed using one-way analysis of variance (ANOVA), with a *P*-value < 0.05 considered statistically significant. All analyses were performed using the STATISTICA program (Version 10; Stat Soft, Inc., 1993), while figures were constructed using the program Sigma Plot (Version14; Systat Software, San Jose, CA; www.systatsoftware.com).

## Results

**Figure 3:**
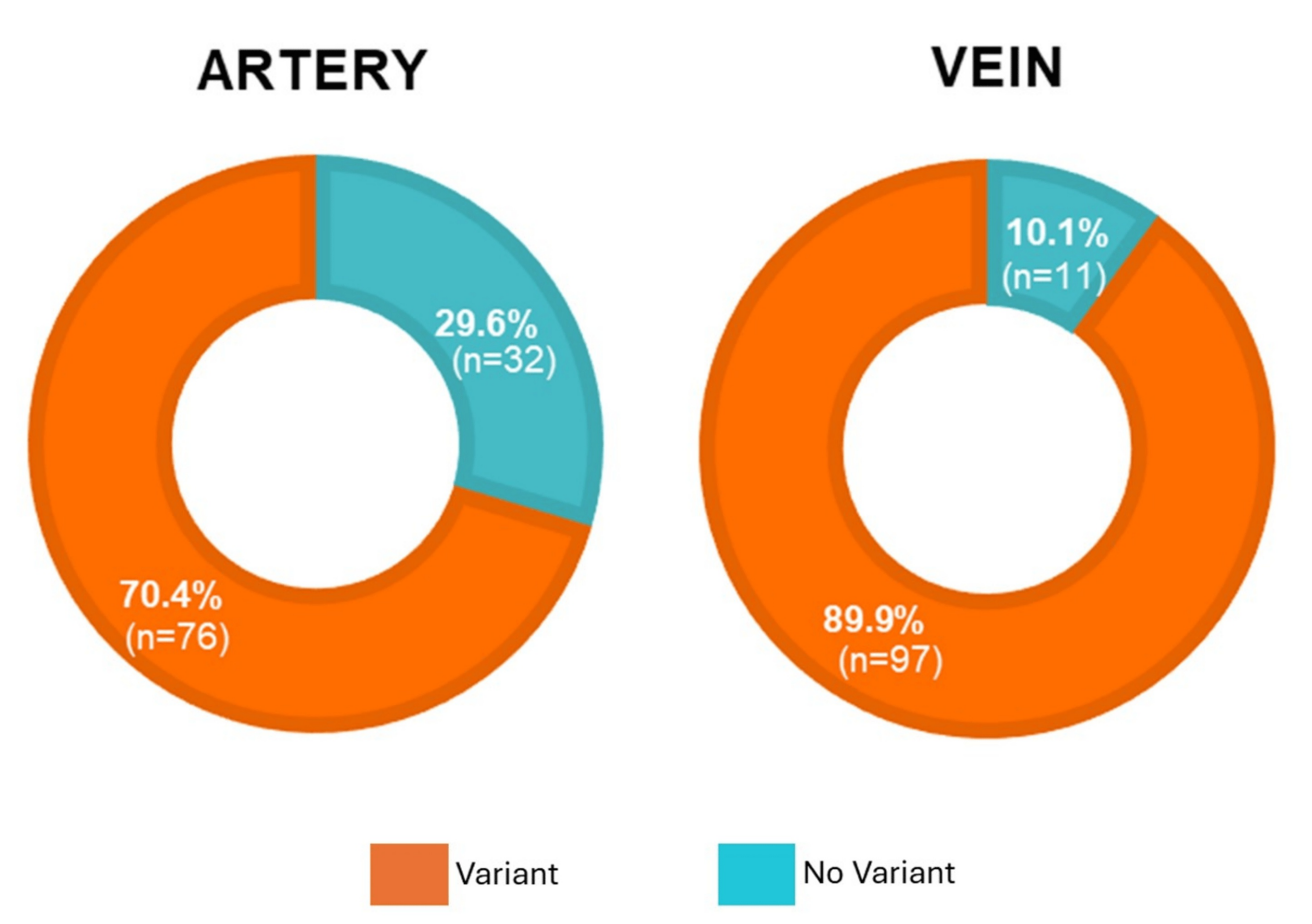
Percentage frequencies of the occurrence of at least one of the three studied variants, corona mortis, aberrant obturator, or accessory obturator, in the arterial or venous pelvic vessels.

Of the 108 hemipelves studied, 14 (13%) were from female cadavers and 94 (87%) from male cadavers, with a mean age of 55.8 ± 2.4 years. We found that 32 (29.6%) of the hemipelves did not present any arterial variants, and 11 (10.1%) did not present a venous variant. Accordingly, the prevalence of arterial variants was 76 (70.4%), and that of venous variants was 97 (89.9%) (Figure 3). Interestingly, we observed the presence of two or more arteries or veins in some hemipelvis that met the definition of one of the three previously defined variants.

**Figure 4:**
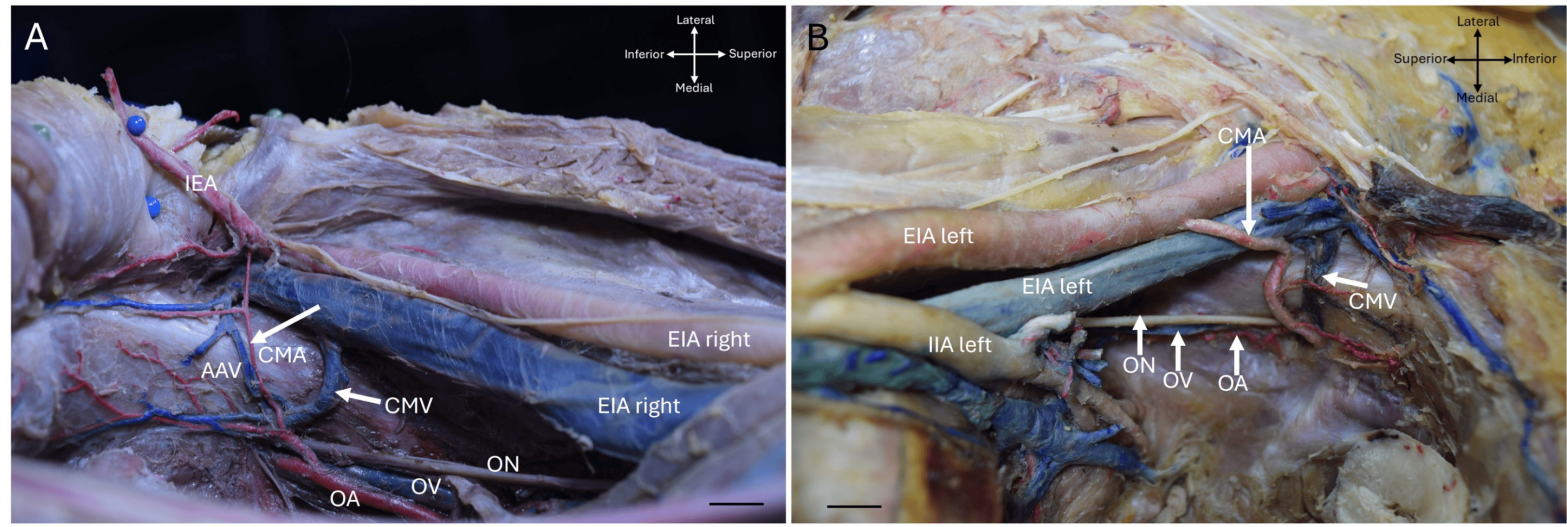
Caliber differences of the corona mortis artery (CMA), indicated by arrows in both panels. The cross-arrows indicate the orientation of the body, and the scale bar represents 1 cm. EIA, external iliac artery; OA, obturator artery; IEA, inferior epigastric artery; AAV, accessory aberrant vein

In the arterial system, we identified 77 arterial variants, of which 37 (48.05%) corresponded to the CM artery. An aberrant obturator artery was present in 39 (50.64%) of cases, and 1 (1.29%) corresponded to an accessory obturator artery. Of the total 77 arterial variants, 75 (97.4%) originated from the inferior epigastric artery, while only 2 (2.59%) originated directly from the external iliac artery (Figure 4A).

**Figure 5:**
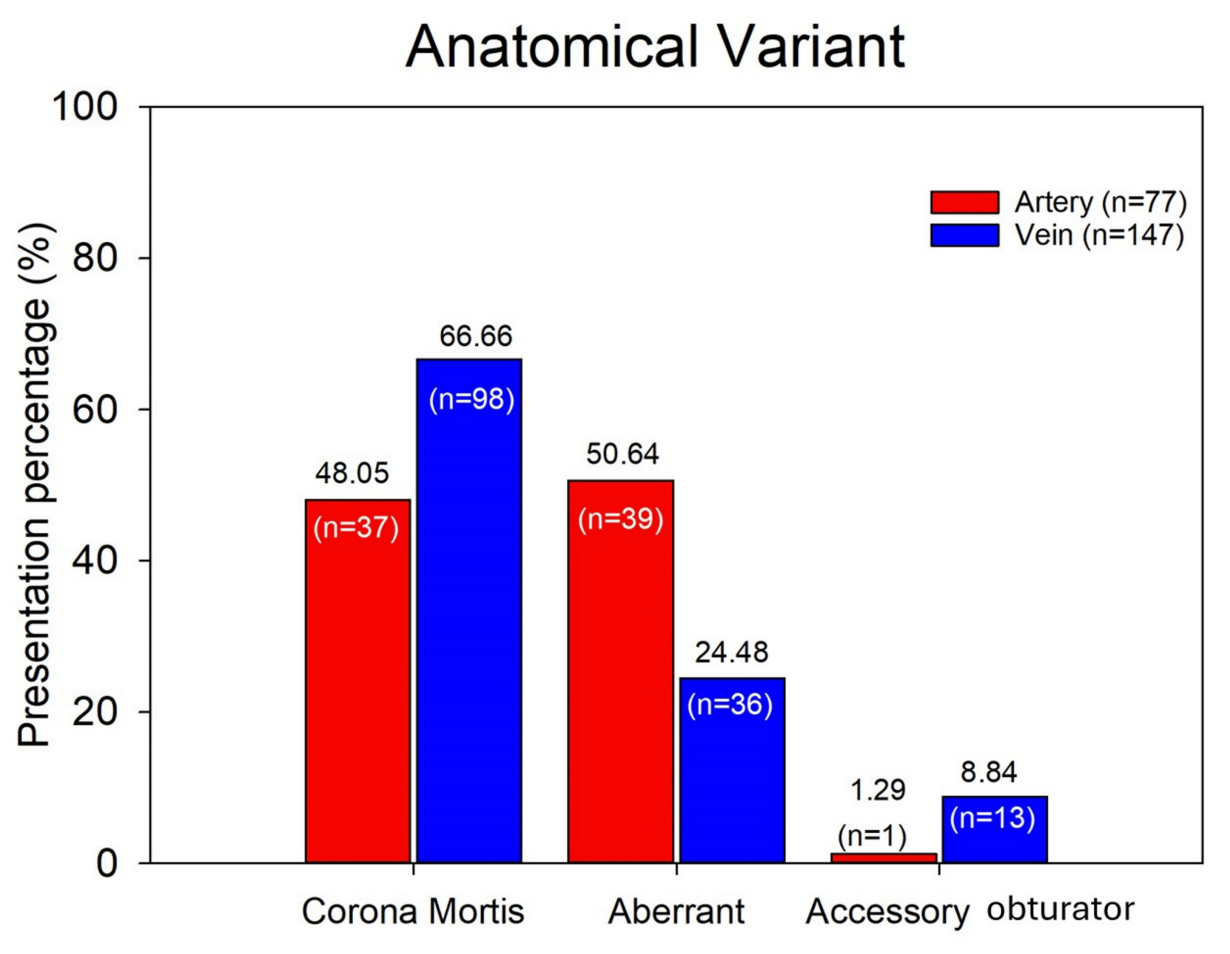
Absolute and percentage frequencies of arterial and venous anatomical variants according to the variation pattern.

In the venous system, 147 veins were identified as variants, distributed as follows: 98 (66.66%) corresponded to CM, 36 (24.48%) to the aberrant obturator vein, and 13 (8.84%) to the accessory obturator vein (Figure 5). Among these veins, 104 (70.74%) drained directly into the external iliac vein, 40 (27.21%) drained into the inferior epigastric vein, and only 3 (2.04%) drained into the common femoral vein.

In the total sample of hemipelves (*n* = 108), CM was found bilaterally, with the artery present in 54 (50%) and the vein in 99 (83.3%) of cases, indicating a higher prevalence of venous CM on both sides of the pelvis. The presence of both arterial and venous CM was identified in 22 hemipelves (20.3%).

Of the hemipelves in which arterial variants were identified, a total of 77 arteries were found, 74 (96%) of which were accompanied by at least one venous variant of equal or larger diameter.

**Figure 6:**
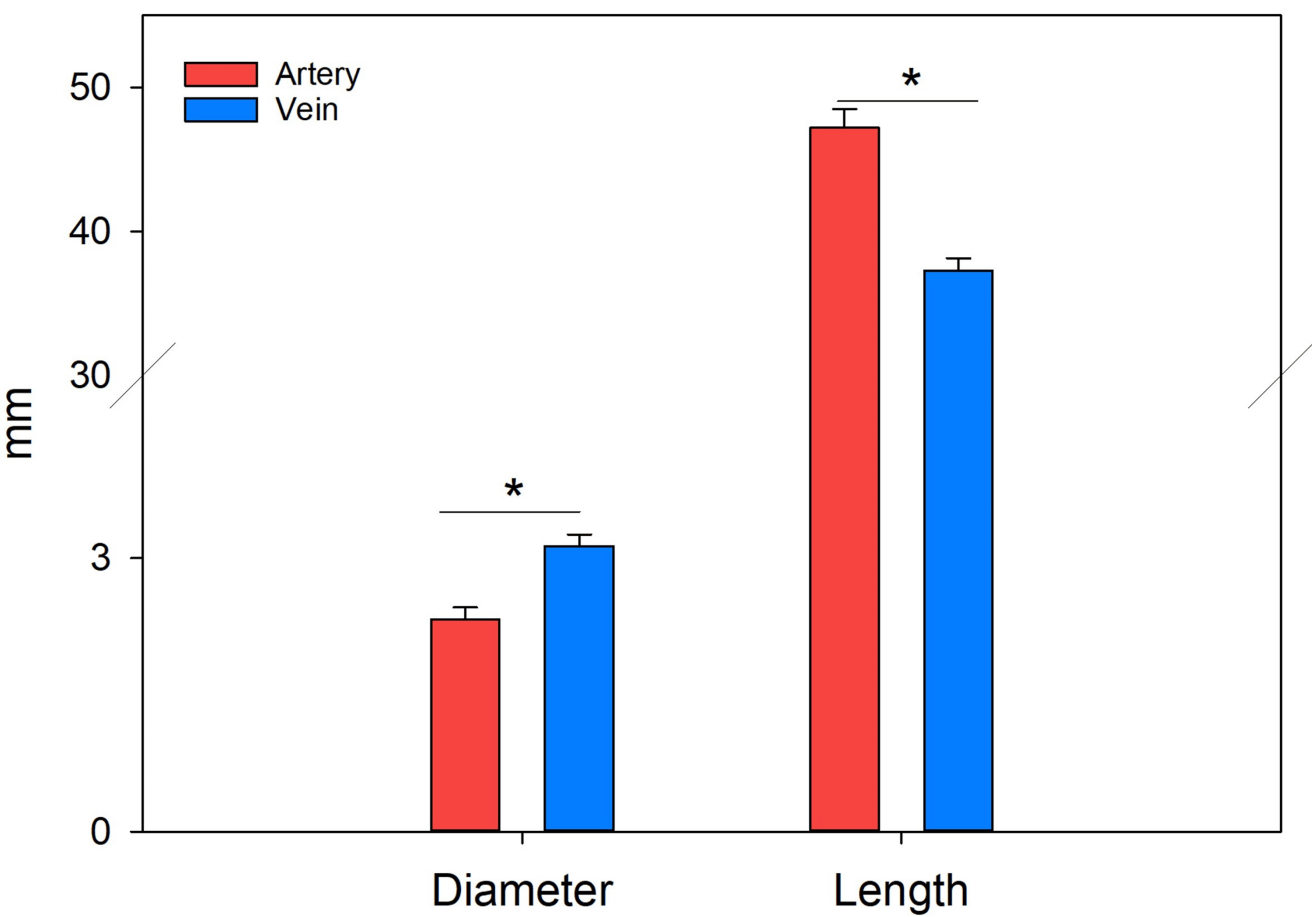
The average diameters and lengths of the arteries and veins of the corona mortis were determined in the study. (*) Statistically significant difference, F(1,33) = 2.058, *P* < 0.00, using a Student’s t-test.

The morphometric analysis of CM revealed a mean arterial diameter of 2.74 ± 0.4 mm and a mean venous diameter of 3.12 ± 0.12 mm (mean ± standard error). One-way ANOVA showed a statistically significant difference, F(1,33) = 2.243, *P* < 0.001. The length of CM from its origin to the entrance of the obturator canal was 47.22 ± 1.29 mm for the artery and 37.29 ± 0.85 mm for the vein, with a statistically significant difference, F(1,33) = 2.058, *P* < 0.001 (Figure 6). Regarding the distance from the CM to the pubic symphysis, the artery measured 51.19 ± 0.81 mm, while the vein measured 55.71 ± 1.12 mm; this difference did not show a statistically significant difference (*P* < 0.08). Similarly, the distance from the anterior superior iliac spine to the CM was 97.86 ± 0.97 mm for the artery and 96.25 ± 0.90 mm for the vein, with no statistically significant difference in one-way ANOVA (F(1,134) = 3.015, *P* < 0.258) (Table 1; Figure 6).

**Table 1:** Morphometric measurements of arteries and veins meeting the anatomical-surgical criteria for the corona mortis.

Corona mortis	Artery	Vein	P
Diameter (mm)	2.74 ± 0.45	3.12 ± 0.12	0.025
Length (mm)	47.22 ± 1.29	37.29 ± 0.85	0.04
Distance (symphysis to corona mortis) (mm)	51.19 ± 0.81	55.71 ± 1.12	0.085
Distance (iliac spine to corona mortis) (mm)	97.86 ± 0.97	96.25 ± 0.90	0.258

## Discussion

The CM artery or vein refers to an anatomical variant in which an anastomosis exists between the obturator artery or vein and the external iliac or inferior epigastric vessels [12]. When present, they are located on the superior border and posterior aspect of the superior pubic ramus [9]. The presence of this anatomical variant has broad clinical implications, as it is closely related to the superior pubic ramus, the acetabulum, and the femoral ring, and therefore represents a significant risk during inguinal or pelvic surgeries [2]. It may also be injured in fractures of the superior pubic ramus, leading to significant bleeding [13]. Likewise, during laparoscopic hernia repair, it is crucial to avoid damaging the ventral preperitoneal space during dissection, as this could compromise the hernial sac [12,14]. CM has also been considered a risk factor in orthopedic surgeries using an anterior approach to the acetabulum [9]. The increasing need for surgical interventions due to trauma, orthopedic procedures on the hip and pelvis, and repair of anterior abdominal hernias requires detailed and adequate knowledge of the morphological and topographical characteristics of CM, as well as the prevalence of this anatomical variant in the treated population.

In a previous study of 80 hemipelvis dissections, the incidence of arterial CM was reported as 36%, and venous CM as 60% [9]. Another study of 204 hemipelves reported CM artery in 22.5%, venous CM in 70.6%, and both structures in 17.2% [15]. Our results, compared with previously reported studies (Table 2), show a higher incidence: 48.05% for arterial CM and 66.6% for venous CM.

**Table 2:** Previously reported patterns of arterial and venous CM in cadaveric studies. CM, corona mortis

Author (Year)	Hemipelvis (*n*)	CM artery (%)	CM venous (%)	CM both (%)
Darmanis et al., 2007 [9]	80	36	60	-
Mahato, 2009 [16]	50	-	40	22
Rusu et al., 2010 [2]	40	65	55	-
Stavropoulou-Deli and Anagnostopoulou, 2013 [7]	20	40	50	-
Leite et al., 2017 [17]	60	45	-	-
Kashyap et al. 2019 [18]	24	8.3	58.3	-
Schaible et al., 2024 [19]	210	22	58	17
Alvarez-Manila et al. (this study)	108	48	66.6	20.3

Furthermore, the presence of both venous and arterial CM in the same hemipelvis is 20%-30%, which is important because our study demonstrates a higher incidence of the CM anatomical variant. Although a previous study reported a bilateral occurrence of venous CM in 26.78% of patients [20], our results indicate a bilateral incidence of venous CM of 80% and a bilateral incidence of 88%, without specifying whether the variant is arterial or venous. It is important to note that the incidence reported in various studies varies due to several factors, one of which is the definition assigned to these variants. Consequently, the reported incidence depends on the definition and classification used by the author, as there is no consensus on defining CM from both anatomical and surgical perspectives.

Clinically and anatomically, the origin of the variant is important. For example, a previous study reported that 26% of CM cases originate from the inferior epigastric artery, 22% from the obturator artery, and the remaining 4% from the external iliac arteries [12].

The latter is important, as a significant incidence of venous CM has been reported, consistently higher than that of arterial CM. Therefore, the probable role of this anatomical structure as a risk factor must be adequately assessed before any surgical intervention [21]. Therefore, given the higher prevalence of venous CM compared to arterial CM, its importance should be considered to prevent venous bleeding. Similarly, the presence of an arterial variant would be suggestive of locating at least one venous variant. A deliberate search for CM in the surgical procedure has been proposed to avoid damaging this vascular network, rather than modifying the surgical procedure [22]. Therefore, CM is an important but manageable vascular variant [23]. In orthopedic surgeries, care must be taken when dissecting near the superior pubic ramus during an anterior approach to the acetabulum, such as the ilioinguinal or intrapelvic (modified Stoppa) approach [9]. Therefore, a thorough understanding of pelvic vascular anatomy makes surgical procedures in this region significantly safer.

Karakurt et al. [24] reported that the distance from CM to the pubic symphysis ranges from 21.4 to 41 mm. Another study showed that the average distance for the artery is 52.4 mm (range, 40-75 mm), while the average distance for the vein is 46.7 mm (range, 35-55 mm) [7]. Based on these findings, we can speculate that perforation of the arterial CM would be more frequent, whereas injury to the venous CM is less likely, as the artery is located closer to the pubic symphysis; however, venous injury depends on the mechanism of trauma.

On the other hand, the average diameter of the arterial CM (2.74 ± 0.4 mm) was smaller than that of the vein (3.12 ± 0.12 mm); however, both diameters are important in terms of hemorrhagic risk during surgical procedures and should not be ignored, even when they fall within previously reported ranges [25].

The presence of a venous CM appears to be common enough to justify its inclusion in the *normal* venous pattern [7,26]. It should be noted, however, that arterial injury is more dangerous in terms of hemorrhage, while venous bleeding is more difficult to identify. Bleeding complications and subsequent laparotomy can result in prolonged hospitalization [5].

Study limitations

Although each measurement was performed in triplicate by three researchers previously trained in the use of the measurement instruments, no statistical reliability tests or inter- or intra-observer error metrics, such as Technical Error of Measurement (TEM), Relative Technical Error of Measurement (rTEM), or reliability coefficient (R), were performed; this is acknowledged as a limitation. However, this does not alter the main finding, which is the high prevalence of CM.

## Conclusions

The study reveals a high prevalence of the CM variant in the study population. Therefore, considering the possibility of identifying a variant of the CM could reduce the rates of bleeding complications during surgical procedures, as adequate anatomical knowledge can provide the advantage of managing and approaching a CM injury with caution.
